# Supplementary material for: Molecular Mechanism of Conformational Crossover of Mefenamic Acid Molecules in scCO2
Source: Materials (Basel). 2023 Feb 7;16(4):1403. doi: 10.3390/ma16041403 (PMC9963762; doi:10.3390/ma16041403)
Supplement: Supplementary file 1 [file materials-16-01403-s001.zip › materials-2146321-supplementary.pdf]

Supplementary Materials

Molecular Mechanism of Conformational Crossover of Mefenamic Acid Molecules in $scCO_2$

Roman D. Oparin *, Mikhail A. Krestyaninov, Dmitry V. Ivlev and Michael G. Kiselev

G.A. Krestov Institute of Solution Chemistry of the Russian Academy of Sciences (RAS), Akademicheskaya St. 1, Ivanovo 153045, Russia

* Correspondence: r.d.oparin@yandex.ru

In our recent works [1–4], we have shown that for drug compounds dissolved in inert solvents, the integral extinction coefficient (ε) of the spectral bands is not a constant value, but linearly (with a small negative slope coefficient α) depends on temperature.

$$\varepsilon(T) = \varepsilon_0 + \alpha(T - T_0) \qquad \text{(S1)}$$

According to the universal light absorption law, the integral intensity of a spectral band is calculated as follows:

$$A(T) = \varepsilon(T) \times c(T) \times l \qquad \text{(S2)}$$

where $c(T)$ is the API molar concentration, l is the optical path length. Expanding the concentration temperature dependence $c(T)$ in a series in the vicinity of temperature T_0, one can obtain the following equation for the integral intensity of the spectral band:

$$A(T) = \left[\varepsilon_0 + \alpha(T - T_0)\right] \times \left[c_0 + \beta_1(T - T_0) + \beta_2(T - T_0)^2 + \beta_3(T - T_0)^3 + \dots\right] \times l \qquad \text{(S3)}$$

As one can see from Eq. 3, the temperature dependence of integral intensity can only be linear when the concentration is constant. Therefore, the linearity of the integral intensity temperature dependence with a negative slope coefficient is an indicator of the solute constant concentration in the phase diagram region under consideration. Indeed, as it follows from our recent works, for true solutions of paracetamol in CCl_4 [1], carbamazepine in tetrahydrofuran [2] and lidocaine in CCl_4 [4] at a constant API concentration, the temperature dependences of the ν(C=O) spectral band integral intensity are linear with a negative slope coefficient (see Figure S1).

Thus, in the present work, for the mefenamic acid solution in $scCO_2$, which contains an API of a certain amount, the linear character of $A(T)$ for the ν(C=O) spectral band (see Figure S1) in the temperature range of 140−180°C unambiguously confirms that the mefenamic acid concentration in the solution is constant.

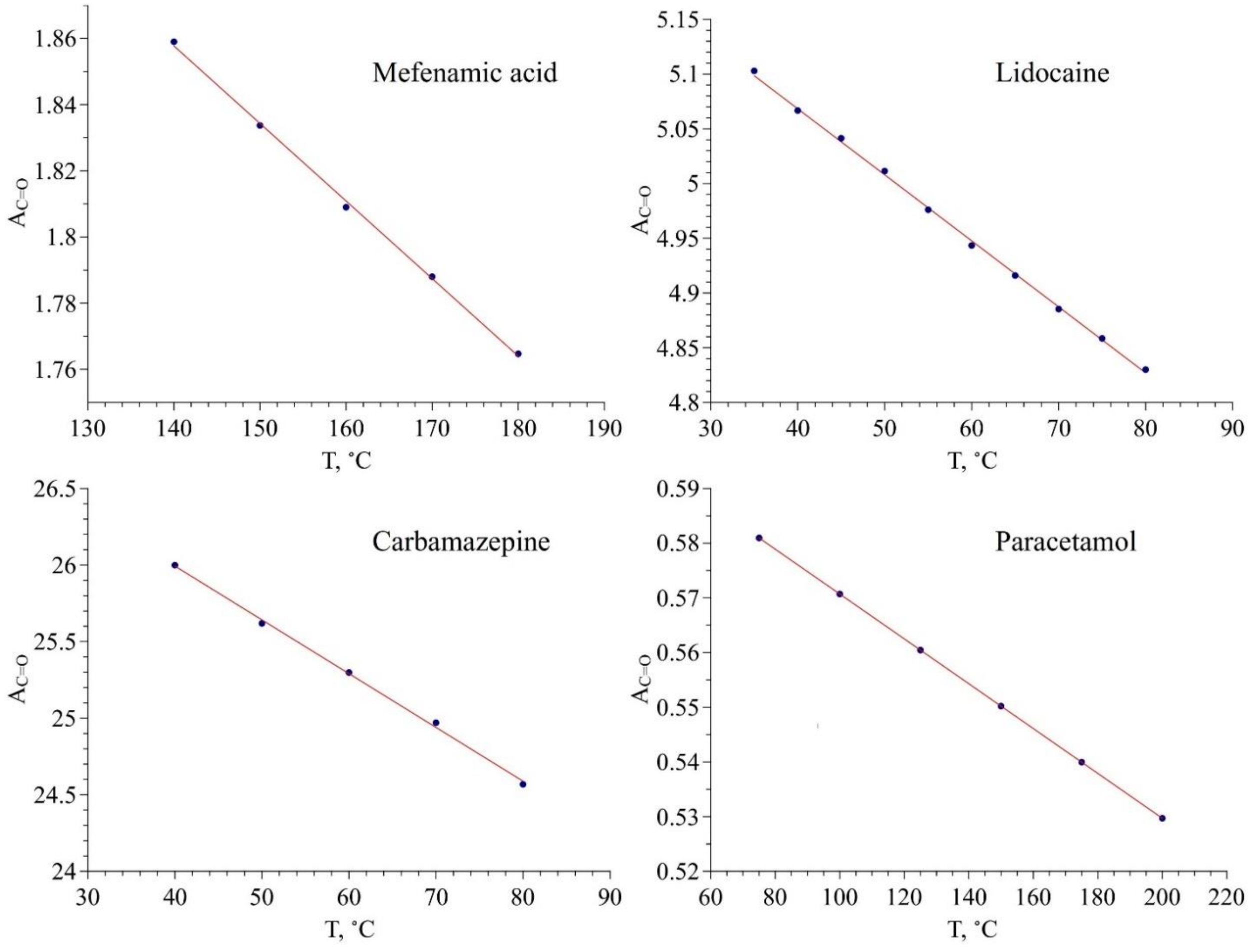

Figure S1. Temperature dependence of the ν(C=O) spectral band integral intensity for mefenamic acid dissolved in $scCO_2$, obtained in the present study as compared to that for true solutions of paracetamol in CCl_4 [1], carbamazepine in tetrahydrofuran [2] and lidocaine in CCl_4 [4].

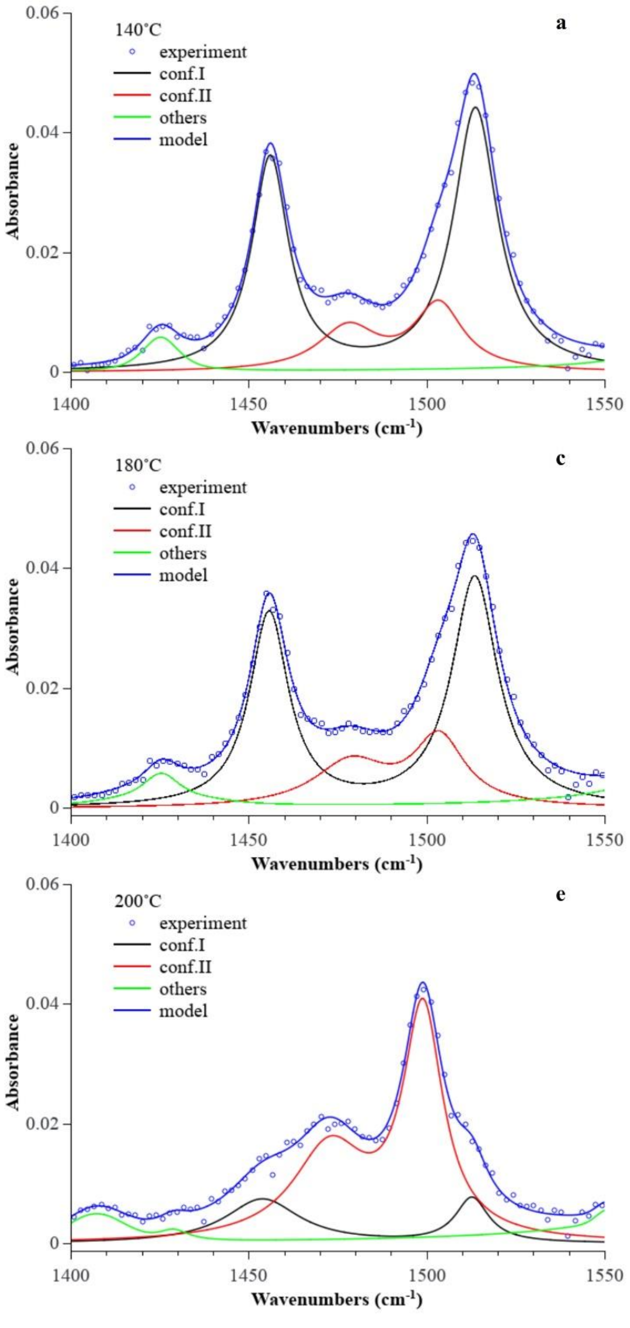

Figure S2. Quality of deconvolution and reproducibility of the experimental spectra of mefenamic acid in scCO_2 in the wavenumber range corresponding to the analytical spectral domain (1400–1550 cm^{-1}). Superposition of two spectral contributions was used for each mefenamic acid conformer. The high-frequency contribution is related to the N–H rocking vibration and the low-frequency one is related to the complex vibrations of the MA molecule aromatic system.

References

1. Oparin, R.D.; Vorobyev, E.A.; Kiselev, M.G. A New Method for Measuring the Solubility of Slightly Soluble Substances in Supercritical Carbon Dioxide. *Russ. J. Phys. Chem. B* **2016**, *10*, 1108–1115, doi:10.1134/S1990793116070149.
2. Kalikin, N.N.; Kurskaya, M. V.; Ivlev, D. V.; Krestyaninov, M.A.; Oparin, R.D.; Kolesnikov, A.L.; Budkov, Y.A.; Idrissi, A.; Kiselev, M.G. Carbamazepine Solubility in Supercritical CO2: A Comprehensive Study. *J. Mol. Liq.* **2020**, *311*, 113104–113110, doi:10.1016/j.molliq.2020.113104.
3. Kalikin, N.N.; Oparin, R.D.; Kolesnikov, A.L.; Budkov, Y.A.; Kiselev, M.G. A Crossover of the Solid Substances Solubility in Supercritical Fluids: What Is It in Fact? *J. Mol. Liq.* **2021**, *334*, 115997, doi:10.1016/j.molliq.2021.115997.
4. Oparin, R.D.; Krestyaninov, M.A.; Kiselev, M.G. Role of an Intramolecular H-Bond in Lidocaine Conformer Distribution and Polymorph Stability. *J. Mol. Liq.* **2022**, *360*, 119461, doi:10.1016/j.molliq.2022.119461.
